# The Role of Doxazosin in PTSD-Related Nightmares: A Case of Comorbid Anorexia Nervosa

**DOI:** 10.1192/bjo.2025.10727

**Published:** 2025-06-20

**Authors:** Amith Gangadharan Remani

**Affiliations:** Queens Hospital, Burton on Trent, United Kingdom

## Abstract

**Aims:** This case involves a 32-year-old female with a history of Anorexia Nervosa and Post-Traumatic Stress Disorder (PTSD) admitted for restricted eating. During admission, she reported worsening PTSD symptoms, including nightmares, linked to a reduction in her doxazosin dose. Doxazosin, an alpha-1 adrenergic antagonist, is used off-label to treat PTSD-related nightmares by reducing noradrenergic hyperactivity. This case highlights the challenges of managing complex comorbidities and balancing physical and mental health.

**Methods:** The patient, a 32-year-old female, was admitted for restricted eating. She had a history of Anorexia Nervosa (restrictive subtype) and PTSD. During admission, she reported increased PTSD symptoms, including frequent nightmares and difficulty distinguishing nightmares from reality.

Initially, her doxazosin dose was reduced from 16 mg to 8 mg due to hypotension concerns. However, this reduction coincided with worsening nightmares and distress. After confirming physical stability (stable blood pressure and no refeeding complications), the dose was increased to 12 mg. This adjustment led to significant improvement: reduced nightmare frequency, decreased distress, and better ability to differentiate nightmares from reality.

Her treatment involved a multidisciplinary approach, including medical monitoring of refeeding syndrome, psychiatric support for PTSD, and nutritional rehabilitation for anorexia nervosa. Regular monitoring of her physical and psychiatric health was maintained throughout her hospital stay.

**Results:** This case illustrates the complex interplay between physical and psychiatric conditions, particularly in patients with comorbid anorexia nervosa and PTSD. The reduction in doxazosin dose likely disrupted its therapeutic effect on PTSD-related nightmares, leading to symptom exacerbation. Doxazosin alleviates nightmares by blocking noradrenergic hyperactivity, which is implicated in PTSD pathophysiology. Restoring the dose to 12 mg balanced psychiatric symptom management with physical stability.

The case underscores the importance of a multidisciplinary approach in managing complex comorbidities. Collaboration between medical, psychiatric, and nutritional teams was essential to address both her physical health (refeeding syndrome, hypotension risk) and psychiatric needs (PTSD-related nightmares, anorexia nervosa). Regular monitoring and individualized treatment adjustments were key to achieving a positive outcome.

This case highlights the need for careful medication adjustments in patients with comorbid conditions. The decision to increase the doxazosin dose was guided by clinical response and physical stability, demonstrating the importance of personalized care.

**Conclusion:** This case demonstrates the challenges of managing comorbid anorexia nervosa and PTSD, particularly when physical and psychiatric symptoms interact. The careful adjustment of doxazosin dose, combined with a multidisciplinary approach, led to significant symptom improvement. It emphasizes the importance of individualized treatment plans, close monitoring, and collaboration between medical and psychiatric teams in achieving optimal outcomes.

